# Multimodality Imaging in Tumor Angiogenesis: Present Status and Perspectives

**DOI:** 10.3390/ijms18091864

**Published:** 2017-08-28

**Authors:** Artor Niccoli Asabella, Alessandra Di Palo, Corinna Altini, Cristina Ferrari, Giuseppe Rubini

**Affiliations:** Nuclear Medicine Unit, Department of Interdisciplinary Medicine, University of Bari “Aldo Moro”, Piazza G. Cesare 11, 70124 Bari, Italy; dipaloalessandra@gmail.com (A.D.P.); corinna.altini@hotmail.it (C.A.); ferrari_cristina@inwind.it (C.F.); giuseppe.rubini@uniba.it (G.R.)

**Keywords:** tumor angiogenesis, radiopharmaceutical, molecular imaging, single photon emission computed tomography (SPECT), (positron emission tomography) PET

## Abstract

Angiogenesis is a complex biological process that plays a central role in progression of tumor growth and metastasis. It led to a search for antiangiogenic molecules, and to design antiangiogenic strategies for cancer treatment. Noninvasive molecular imaging, such as positron emission tomography (PET) and single photon emission computed tomography (SPECT), could be useful for lesion detection, to select patients likely to respond to antiangiogenic therapies, to confirm successful targeting, and dose optimization. Additionally, nuclear imaging techniques could also aid in the development of new angiogenesis-targeted drugs and their validation. Angiogenesis imaging can be categorized as targeted at three major cell types: (I) non-endothelial cell targets, (II) endothelial cell targets, and (III) extracellular matrix proteins and matrix proteases. Even if radiopharmaceuticals studying the metabolism and hypoxia can be also used for the study of angiogenesis, many of the agents used in nuclear imaging for this purpose are yet to be investigated. The purpose of this review is to describe the role of molecular imaging in tumor angiogenesis, highlighting the advances in this field.

## 1. Introduction

The development of new blood vessels from pre-existing vascular beds is called angiogenesis, and is an important process for tumor growth, induced by the request of oxygen and nutrients [1,2]. In the absence of neovascularization, cancer cells stop expanding, and consequently, the inhibition of angiogenesis may even result in tumor regression, as shown in various experimental models [2,3,4]. Furthermore, neo-angiogenesis promotes the dispersion of tumor cells and metastasis; for this reason, antiangiogenic drugs could slow or even stop tumor growth and prevent metastasis [5,6].

The regulation of angiogenesis includes numerous molecular pathways that involve several mediators, such as hypoxia-inducible factor 1 (HIF-1), growth factors/growth factor receptors like (vascular endothelial growth factor (VEGF), platelet-derived growth factor (PDGF) and fibroblast growth factor-2 (FGF-2)), matrix metalloproteinases (MMPs), αvβ3 integrin, and E-selectin. These molecular pathways can be considered as potential targets for diagnostic and therapeutic interventions [1,7,8,9]. Despite the existence of various angiogenesis-stimulating factors, VEGF is considered the most potent and predominant factor [7,10]. Integrins have also been implicated in a number of processes related to angiogenesis, including cell adhesion, migration, proliferation, differentiation, and survival [11].

Several agents against angiogenesis have even been approved for therapeutic use in cancer patients, but it is very difficult to evaluate the treatment response of these anti-angiogenesis drugs, because of their cytostatic, rather than cytotoxic, effect.

Computed tomography (CT) and magnetic resonance imaging (MRI) may not be suitable for assessing the response to anti-angiogenic treatment. In fact, these techniques only evaluate parameters such as changes in tumor volume or morphology [12].

New technologies, like dynamic contrast-enhanced CT, MRI, or ultrasound, can be used to measure vascular permeability, and tumor blood flow and blood volume, but they cannot measure changes in tumor vascularity [12]. On the contrary, molecular imaging seems to have an important impact on drug evaluation and development [13]. Non-invasive molecular imaging, such as positron emission tomography (PET) and single photon emission computed tomography (SPECT), can allow earlier diagnosis and better prognosis, which will eventually allow for personalized molecular medicine [14]. ^18^F-fluorine-labeled fluorodeoxyglucose (^18^F-FDG) PET/CT has been widely used in oncology for diagnosis, staging, restaging, and monitoring of the therapies’ efficacy. This technique is based on the preferential uptake of the tracer by tumors having a high glucose metabolic activity [15]. However, some studies that used ^18^F-FDG observed that this tracer is not the most suitable radiopharmaceutical for evaluating the angiogenesis, because it did not show significant change in tumor tracer uptake [16].

Several authors have studied new radiopharmaceuticals specific for tumor angiogenesis, in particular, potential targets for VEGF, αvβ3 integrin, fibronectin, and MMPs (Table 1).

## 2. ^18^F-FDG-Based Imaging

The role of ^18^F-FDG PET/CT in predicting tumor angiogenesis and evaluating the response to anti-angiogenic treatment is uncertain. Several studies describing a correlation between angiogenic activity in tumors and ^18^F-FDG uptake, in vitro and in vivo, are reported in the literature. Furthermore, there is evidence that the stabilization of HIF is able to modulate tumor angiogenesis and increase glucose metabolism [17]. Pedersen et al. investigated the association of glucose transporters and vascular endothelial growth factor (VEGF) in two human small-cell lung cancer lines. Authors evaluated changes in the expression of glucose transporters (GLUTs) and VEGF during 12-, 18-, and 24 h of severe hypoxia in vivo (xenografts) and in vitro (cell cultures), and demonstrated co-upregulation of both GLUT-1 and VEGF, which suggests a modulation of the glucose kinetics by angiogenesis-related genes [18]. Airley and Mobasheri considered the pathways related to hypoxic regulation of glucose transport, metabolism, and angiogenesis, and highlighted the link between hypoxia, angiogenesis, and glucose transporters [19].

In reference to non-small cell lung cancer, some studies show good associations between ^18^F-FDG PET uptake and tumor angiogenesis. Guo et al. investigated the correlation between microvessel density (MVD) and ^18^F-FDG uptake using immunohistochemical staining measurements of angiogenesis with antibodies to CD105. MVD is a proliferation-related endothelial cell marker that reflects active angiogenesis, positively correlates with ^18^F-FDG uptake, and is a good indicator of prognosis in lung adenocarcinomas [20]. On the contrary, Cherk et al., demonstrated no significant correlation in non-small cell lung cancer between hypoxia and glucose metabolism assessed by ^18^F-FDG [21]. Strauss et al. examined 25 patients with colorectal cancer, and concluded that angiogenesis-related gene expression is a determining factor in ^18^F-FDG kinetics [22]. Recently, Groves et al. observed in 20 patients with early breast carcinoma, a correlation among maximum standardized uptake value (SUVmax), mean standardized uptake value (SUVmean), and CD105; in particular, SUVmean appeared to be associated with immunohistochemical metabolic markers [23].

**Figure 1:**
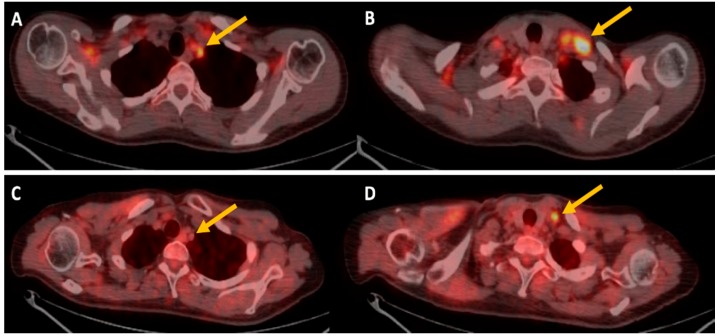
^18^F-FDG PET/CT imaging of a patient treated with bevacizuamb. (**A**,**B**) are transaxial PET/CT images at baseline that shows high ^18^F-FDG uptake in left supraclavicular lymph nodes (yellow arrows), (**C**,**D**) transaxial PET/CT images after therapy shows reduction of ^18^F-FDG uptake (yellow arrows).

Finally, considering that the response to anti-angiogenic therapy may be inadequately assessed by traditional size-based radiological criteria, several authors evaluated the role of ^18^F-FDG PET/CT. De Bruyne studied patients with metastatic colorectal cancer, and demonstrated that SUVmax, complete metabolic response, and low MVD, are favorable prognostic factors after neoadjuvant chemotherapy with bevacizumab [24]. Recently, Hwang et al. have focused on the PET/CT semi-quantitative parameters (metabolic tumor volume and total lesion glycolysis) that seem to be good prognostic factor safer treatment with anti-VEGF targeted agents [25] (Figure 1).

## 3. VEGF/VEGFR Pathway and Radionuclide-Based Imaging

Several recent studies have focused on radiopharmaceuticals that target the angiogenesis pathway mediated by VEGF and its receptor tyrosine kinase (VEGFR) [26,27,28]. During tumor growth, the oxygen deficiency (hypoxia) represents the pro-angiogenic signaling mediated by VEGF/VEGFR [29,30,31]. The VEGF actions are mainly mediated by 2 endothelium-specific tyrosine kinases receptors, VEGFR-1 and VEGFR-2 [32]. VEGFR-2 is the major mediator of angiogenesis, is overexpressed in a variety of solid tumor cells, and it is considered a poor prognostic marker for the survival of cancer patients [7,33,34]. The binding of VEGF to its receptor initiates a signaling cascade that promotes the proliferation, migration, and survival of endothelial cells, ultimately leading to angiogenesis [35,36]. Therefore, new therapies based on humanized monoclonal antibodies (such as bevacizumab) inhibiting the isoform VEGF-A are used to treat colorectal, lung and ovarian cancer. Other newer therapies are under various research stages that lead to a greater interest around VEGF/VEGFR radiopharmaceuticals [37,38].

VEGF/VEGFR imaging was achieved with SPECT and PET, and several radioisotopes, such as ^123^I, ^111^In, ^99^mTc, ^64^Cu, and ^89^Zr, were used. In order to explore the possibility of VEGFR scintigraphy of primary tumors and their metastasis, some authors analyzed the binding properties of ^123^I-labelled VEGF165 (^123^I-VEGF165) and ^123^I-VEGF121 (that are the predominant VEGF human isoforms) to human umbilical vein endothelial cells, several human tumor cell lines, a variety of primary human tumors, and some adjacent non-neoplastic tissues, as well as normal human peripheral blood cells in vitro. Not only did they demonstrate the existence of specific binding sites for ^123^I-VEGF165 and ^123^I-VEGF121 in human endothelial cells, but also, in several tumor cells that express significantly higher numbers of VEGF receptors compared to corresponding normal tissues. Differently from ^123^I-VEGF121, ^123^I-VEGF165 binds to more types of tumor cells and primary tumors with higher binding capacity. The study conducted by Li et al. provides the basis for further studies regarding in vivo localization and diagnosis of solid tumors, and their metastasis using radiolabeled VEGF165 [39]. In fact, it has led the same authors to develop an ^123^I-VEGF165 receptor scintigraphy, to explore a possible role of VEGF receptor scintigraphy in the staging and follow-up of patients with solid tumors; in particular, in 18 patients with gastrointestinal tumor,^123^I-VEGF165 scans were compared with CT and MRI, demonstrating the usefulness of the ^123^I-VEGF165 scan to visualize the tumor angiogenesis, despite the superiority of CT and MRI for the visualization of the gastrointestinal tumors and metastasis [40]. Li et al. further investigated biodistribution, safety, and dosimetry of ^123^I-VEGF165 in 9 patients with pancreatic carcinomas; ^123^I-VEGF165 scans visualized the primary pancreatic tumor and their metastasis, but also the thyroid which appeared to be the organ with the highest absorbed dose due to severe deiodination [41].

Yoshimoto et al. labeled VEGF121 and VEGF165 with ^125^I and compared them; interestingly, ^125^I-VEGF121 accumulation in tumors decreased with increasing tumor volume, suggesting that small tumors have higher VEGFR expression than larger tumors. It was also found that ^125^I-VEGF165 uptake was higher than ^125^I-VEGF121 uptake in some organs (such as the kidneys, heart, and lungs) but lower in many others; the authors concluded that ^125^I-VEGF121 is a promising tracer for noninvasive delineation of angiogenesis in vivo [42].

In preclinical studies, VEGF121 has also been labeled with ^99^mTc, and the stability of this tracer in a murine mammary carcinoma model was evaluated. The authors measured the tumor uptake of ^99^mTc-VEGF121 as percentage of the injected dose per gram of tissue (expressed as %ID/g), suggesting that ^99^mTc-VEGF121, stable for about 1 hour in vivo, can be used to visualize mouse tumor neovasculature in millimetric lesions [43,44]. In another study by Blankenberg et al., this tracer was also applied to evaluate the tumor vasculature before and after chemotherapy, in particular, they demonstrated that it can reveal the heterogeneity of tumor vasculature in an orthotopic mouse model of mammary adenocarcinoma, and its response to low-dose metronomic (antiangiogenic) and high-dose (tumoricidal) cyclophosphamide treatment [45].

Chan et al. studied^111^In labeled with a recombinant protein composed of VEGF165 linked to human transferrin (hnTf-VEGF) without DTPA as metal chelator, that interacted specifically with VEGFR, but not with transferrin receptors. The authors evaluated the localization properties of ^111^In-labeled hnTf-VEGF in the tumor and normal tissues of athymic mice implanted subcutaneously with highly vascularized glioblastoma xenografts. ^111^In-hnTf-VEGF seems to be a promising radiopharmaceutical for imaging tumor angiogenesis [46].

Cai et al. labeled several VEGF121 isoforms with ^64^Cu for PET imaging. The limit of these tracers was the high VEGFR-1 expression in the kidney, and consequently, the toxicity in it; subsequently, ^64^Cu-DOTA-VEGF121 (DEE), a mutant VEGF121 specific for VEGFR2, has been developed, demonstrating lower kidney toxicity. The PET imaging of small animals revealed rapid, specific, and prominent uptake of this ^64^Cu-DOTA-VEGF121 (DEE) in highly vascularized small tumors, with high levels of VEGFR-2 expression, but significantly lower and sporadic uptake in large tumors, with low levels of VEGFR-2 expression. This study demonstrated the dynamic nature of VEGFR expression during tumor growth, in fact, in the same tumor model, levels of VEGFR expression were different at different sizes and stages [47,48].

Other studies are about radioisotopes labeled to anti-VEGF human antibodies, such as bevacizumab, a drug that blocks and neutralizes VEGF. In a study by Nagengast et al., ^111^In and ^89^Zr labeled to bevacizumab were developed respectively for SPECT and PET imaging and to visualize and quantify VEGF in vivo.^89^Zr-bevacizumab, ^111^In-bevacizumab, or ^89^Zr-Immunoglobulin (IgG) were injected into micexenografted with human ovarian tumors. The tumor uptake of ^89^Zr-bevacizumab and ^111^In-bevacizumab resulted significantly higher compared to tumor uptake of the control ^89^Zr-IgG. Furthermore, ^89^Zr-bevacizumab and ^111^In-bevacizumab had a high tumor uptake after 24 hours from injection and a good tumor to background ratio after 72 h. These results show that radioisotopes labeled to bevacizumab are useful for in vivo evaluation of VEGF [49]. Oosting et al. determined tumor uptake of ^89^Zr-bevacizumab in metastatic renal cell carcinoma patients before and during anti-angiogenic therapy, concluding that high baseline tumor SUVmax was associated with longer time to progression [50].

The imaging of VEGFR expression in anti-VEGFR cancer therapy has an important role because the treatment efficacy may vary among various tumor types. The evaluation of VEGFR expression with noninvasive imaging can be useful in the choice of a potentially more effective treatment. Although radiolabeled VEGF isoforms showed a good binding capability for VEGFRs, their in vivo stability, pharmacokinetics and target affinity, are yet to be improved.

## 4. Integrin αvβ3Pathway and Radionuclide-Based Imaging

Integrins are heterodimeric glycoprotein with adhesive capacity, composing by 2 transmembrane subunits (α and β), paired thanks to a large extracellular segments [77]. Also, integrin signaling is essential in tumor angiogenesis and metastasis. In fact, during tumor angiogenesis, integrins expressed on endothelial cells control cell migration and survival, while during metastasis spread, integrins expressed on tumor cells facilitate invasion and movement across blood vessels [78]. Among all integrins, integrin αvβ3 is significantly up regulated on tumor vasculature, but not on quiescent endothelium; this subtype binds to arginine–glycine–aspartic acid (RGD) containing components of the extracellular matrix. Furthermore, many monoclonal antibodies, cyclic RGD peptide antagonists, and peptidomimetic agents against integrin αvβ3 have been used for anti-angiogenic cancer therapy [78,79,80].

Since integrin αvβ3 have an important role in tumor growth and spread, imaging of integrin αvβ3 expression with PET can be useful to evaluate patient risk and to select a target anti-angiogenic therapy. In preclinical studies, the integrin αvβ3 expression seems to be related to tumor aggressiveness and metastatic potential in malignant tumors. For example, integrin αvβ3 plays a role in malignant melanoma, during the transition of cells from the radial growth phase to the vertical growth one [81,82].

Most integrin-targeted imaging tracers have tripeptide Arg–Gly–Asp (RGD) acid sequences (RGD peptides) as the targeting ligands, because of their high affinity and specificity for integrin αvβ3. The first RGD-based tracers were described in 1999; they were SPECT tracers, radiolabeled with ^125^I, and used for imaging integrins in three different mice tumor models (melanoma, mammary carcinoma, and osteosarcoma) [51]. Since then, also RGD peptide based PET tracers have been developed and among these ^18^F-galacto-RGD was the first used in humans. Subsequently others tracers such as ^18^F-fluciclatide, ^18^F-RGD-K5, ^18^F-FPPRGD2, ^18^F-alfatide, ^68^Ga-NOTA-RGD and ^68^Ga-NOTA-PRGD2. RGD PET tracer have been studied [51,52,53,54,55,56]. 

In the first study, Haubner et al. demonstrated that the ^18^F-galacto-RGD uptake in the tumor correlates with αvβ3 expression, subsequently determined by Western blot analysis, using a small-animal PET scanner. Furthermore, they studied 9 patients affected by melanoma or sarcoma, both with ^18^F-FDG PET and ^18^F-galacto-RGD, and concluded that ^18^F-galacto-RGD can be applied to assess successful blocking of αvβ3 integrin by therapeutic agents [56]. In another study, Beer et al. performed ^18^F-galacto-RGD PET in 19 cancer patients, demonstrating a highly favorable biodistribution in humans with specific receptor binding, in particular, with high variations of tumor uptake in melanoma patients. Authors concluded that ^18^F-galacto-RGD allows visualization of αvβ3 expression in tumors with high contrast, and confirmed that this tracer offers a new strategy for noninvasive monitoring of molecular processes and may supply helpful information for planning and controlling of therapeutic approaches targeting the αvβ3 integrin [57].

In a recent review, Chen et al. compared radio synthesis, dosimetry, pharmacokinetics, and clinical efficacy of the clinically available RGD-based PET tracers. About radio synthesis, all ^18^F-labeled RGD peptides were subjected to a multi step and time-consuming process while the synthesis with ^68^Ga takes shorter times, even if the short half-life (68 min) of ^68^Ga makes its commercial distribution difficult. Regarding dosimetry, regarding dosimetry, all tested RGD PET tracers are safe and those labeled with ^18^F are comparable to ^18^F-FDG (effective doses range from 10–40 µSv/MBq). About pharmacokinetic properties, all investigated RGD peptides are very similar in vivo, although structurally different; the elimination is predominantly renal, with important tracer uptake in kidneys and bladder. Considering the in vivo biodistribution, RGD PET tracers are well suited for detecting lesions in lungs, mediastinum, head-and-neck area, breast, and skeletal system; they demonstrate better detection efficiency for cancers with low or intermediate ^18^F-FDG uptake (prostate cancers, carcinoid tumors), and for brain tumors. In particular, they may have higher sensitivity and specificity respectively for identifying glioma and for defining tumor boundary. Furthermore, RGD uptake and tumor differentiation correlated positively in sarcoma and glioma. About clinical efficacy, RGD PET tracers are useful for tumor detection and staging [55,56,57,58].

In a recent prospective clinical study, 12 patients with brain glioma diagnosed by MRI, underwent ^68^Ga-PRGD2 PET/CT and ^18^F-FDG PET/CT scans before surgery. The expression of integrin αvβ3, CD34, and Ki-67 was determined by immunohistochemical staining of the resected brain tumor tissue. Authors demonstrated that ^68^Ga-PRGD2 PET/CT is a specific method for identifying and assessing glioma neovasculature formation and glioma cells in patients with glioma. The SUVmax of ^68^Ga-PRGD2 is significantly correlated with glioma grading, and the target background ratio maximum (TBRmax) of ^68^Ga-PRGD2 is superior to ^18^F-FDG for differentiating the grading. Furthermore, ^68^Ga-PRGD2 PET/CT may be a useful tool for assessing glioma demarcation and neovasculature formation [60].

Other authors studied ^64^Cu as radionuclide for labeling of RGD-peptides [61]. Thus, a variety of tracers labeled with this isotope have been developed. In one study, a DOTA-conjugated RGD peptide (DOTA-RGDyK) labeled with ^64^Cu was proposed, this tracer demonstrated a highest activity concentration in liver, intestine, and bladder; for this reason it needs further optimization [62]. Others radioisotopes, ^111^In and ^68^Ga, were used to labeled another DOTA-derivatized RGD peptide (DOTA-RGDfK). Studies conducted in αvβ3-positive melanoma model reported that ^111^In and ^68^Ga radiolabeled peptides had specific binding to αvβ3, similar to ^18^F-galacto-RGD. However, ^68^Ga- DOTA-RGDfK demonstrated a higher interaction with proteins in the blood, a higher blood pool activity in vivo, and thus, a lower tumor to background ratios compared to ^18^F-galacto-RGD [59].

## 5. Fibronectin and Matrix Metalloproteinasis Pathway and Radionuclide-Based Imaging

Another potential target for radionuclide-based imaging that can evaluate tumor angiogenesis focus on antibodies/proteins targeting on single-chain Fv antibody fragments specific binding to a fibronectin isoform. There are several fibronectin isoforms (e.g., III CS, ED-A, ED-B) that participate to cell migration, oncogenic transformation and other subsequent processes. The ED-B domain isoform is the most present in fetal and neoplastic tissues, while it is less present in normal adult tissues, therefore being an important marker for angiogenesis, due to its involvement in vascular proliferation [83,84].

Based on these findings, several radionuclides labeled with anti-ED-B antibody fragment have been developed. Recombinant and chemically modified derivatives of the single-chain antibody fragment (scFv) L19 specific for the ED-B fibronectin isoform, have been labeled with ^99^mTc and used in tumor bearing mice, providing a potentially useful clinical tool for angiogenesis imaging [63]. In a pre-clinical study, Tarli et al. described the distribution of the ED-B containing fibronectin in four different tumor animal models, and the tumor-targeting properties of a radiolabeled anti-ED-B antibody fragment; they reported the possibility to selectively target tumoral vasculature using the human recombinant antibody (scFv) L19 [64]. In another study, Santimaria et al. studied (scFv) L19 radiolabeled with ^123^I in 20 patients with brain, lung, or colorectal cancer. In particular, they reported interesting results in anaplastic astrocytoma, because it widely expresses ED-B; so, this non-invasive method can provide follow up information about tumors that may switch from low grade to anaplastic [65].

All these observations indicate that radiolabeled antibody fragments against the ED-B domain of fibronectin offer a number of important prospects as potential new tracers for non-invasive angiogenesis imaging and for therapies, with the possibility to develop therapeutic radionuclides or toxic agents that are selective to tumoral vasculature [85,86]. Other authors have analyzed imaging of the ED-B domain of fibronectin using PET isotopes, such as ^76^Br and ^124^I, which labeled always to antibody fragments; the potential use of ^124^I-L19-SIP appears very interesting, not only for immuno-PET imaging of tumor angiogenesis, but also as a guide for ^131^I-L19-SIP radio-immunotherapy [66,67]. However, further studies are necessary to confirm its appropriateness and usefulness.

Another pathway of neoangiogenesis involves matrix metalloproteinases (MMPs), which are proteolytic enzymes produced after the activation of the endothelial cells that have both a pro-angiogenic and anti-angiogenic role. Their ability to degrade the basal membrane and the extracellular matrix (ECM) provides space for the sprouting vessels and releasing matrix-bound proangiogenic factors, as well as cleaving matrix components into anti-angiogenic factors [87,88]. There are five different classes of MMPs, and they include the gelatinases MMP-2 and MMP-9, that are overexpressed in neoplastic tissue, and are correlated with tumor aggressiveness and metastatic potential; MMPs are also potential targets for therapeutic interventions [89,90,91,92].

In a preclinical study by Furomoto et al., the ^18^F-labelled MMP-2 inhibitors’ (^18^F-SAV03M) role was evaluated, with results that suggest these radiopharmaceuticals as potential and suitable tracers for tumor imaging with PET [68]. These MMP-2 inhibitors are also labeled with 11C-labelled in other pre-clinical studies, showing strong inhibitory effectiveness for the gelatinases MMP-2 and MMP-9 [93]. Recently, both ^18^F-NOTA and ^68^Ga-NOTA labeled to C6 (another selective gelatinase inhibitor) have been studied as potential radiopharmaceuticals for the imaging of in vitro MMP2 activity in tumor models [69].

## 6. Radiopharmaceuticals for Hypoxia Imaging

Tissue hypoxia is the result of imbalance between oxygen supply and consumption that led to inadequate tissue oxygenation. Hypoxia in malignant tumors can affect the treatment outcome, in fact in lack of oxygen, malignant tumors are relatively resistant both to chemotherapy and radiotherapy. Another pathogenetic factor that caused hypoxia is related to the chaotic and primitive tumor microvasculature, characterized by structural and functional abnormalities, and heterogeneous microcirculation patterns; these characteristics represent a limit for oxygen diffusion [94]. The cellular response to hypoxia is mainly controlled by the family of hypoxia-inducible factors (HIFs). The main HIF family member is HIF-1, a heterodimeric protein consisting of two subunits: α-subunit, that is oxygen responsive; and β-subunit, that is constitutively expressed. In the presence of oxygen, HIF-1α is continuously synthesized and degraded, but when oxygen is lacking, the protein accumulates and acts as a transcription factor to up regulate a multitude of genes, including those involved in angiogenesis [95]. At the molecular level, HIF-1 binds the hypoxia response elements (HRE) that induce the up regulation of genes including VEGF, glycolytic enzymes, glucose transporters (GLUT-1), and insulin-like growth factors [96,97]. Several retrospective immunohistochemical studies have demonstrated that hypoxia-mediated expression of HIF-1α is a negative prognostic indicator for many cancer types [98]. Hypoxia-induced changes in tumor behavior seem to create a favorable environment for tumor progression, development of metastases, and therapy-resistant clones; this hypoxia-induced metastatic phenotype may be one of the reasons for the failure of anti-angiogenic drugs [99,100].

^18^F-fluoromisonidazole (^18^F-FMISO) PET/CT has been validated as an effective method of imaging hypoxia, and can capture hypoxic tissues by selectively taking an analog of nitroimidazole; in lack of oxygen,^18^F-FMISO is reduced and covalently bound to intracellular macromolecules, and cannot exit the hypoxic cells; in this way, ^18^F-FMISO measures the degree of intracellular hypoxia in cancer cells. It is the most extensively hypoxia biomarker studied with PET imaging [70,71,72]. Ueda et al. studied the therapeutic effect of bevacizumab in breast cancer using ^18^F-FMISO PET/CT; authors suggested that bevacizumab treatment could have negative effects in some patient, such as shortening survival, by triggering hypoxia and promoting cancer progression. In this study, the ^18^F-FMISO PET/CT scans showed that non-responding tumors treated with bevacizumab exhibited significantly higher ^18^F-FMISO SUVmax at baseline, and after the second course of chemotherapy, than responding tumors. Based on this theory, quantitative and continuous measurements of tumor vascular remodeling and hypoxia in clinical practice are necessary to monitor the therapeutic response in terms of the anti-angiogenic strategy [73]. Recently Bekaert et al. have investigated the relationship between the uptake of ^18^F-FMISO, and other markers of hypoxia and angiogenesis, with patient survival. They studied 33 glioma patients with ^18^F-FMISO PET/CT before surgery, and evaluated biomarkers of hypoxia and angiogenesis with immunohistochemistry on tumor specimens. Authors demonstrated that: (1) the expression of these biomarkers was higher in patient with positive ^18^F-FMISO PET/CT, (2) a correlation between ^18^F-FMISO uptake and the expression of HIF-1α and VEGF existed, and (3) negative ^18^F-FMISO PET/CT patients had a longer survival time than the positive ones [74].

A complex of Cu with diacetyl-bis (N4-methylthiosemicarbazone) (ATSM) ligands is an alternative tracer to study hypoxia with PET imaging; this complex could be labeled with copper positron emitter radioactive isotopes, like ^60/61/62/64^Cu. Cu-ATSM is lipophilic, and with low molecular weight, thus rapidly get into cells thanks to its high membrane permeability. Intracellular Cu-ATSM undergo to a reduction of Cu(II) to Cu(I), followed by re-oxidation thanks to intracellular molecular oxygen. During hypoxia, the unstable Cu(I)-ATSM complex may dissociate into Cu(I) and ATSM, and Cu(I) ion is trapped into cells, while in the presence of oxygen, the [Cu(I)-ATSM] can be re-oxidized into Cu(II)-ATSM, thus allowing efflux from the cell [75].

In a review, Bourgeois et al. compared ^18^F-FMISO and Cu-ATSM, concluding that both ^18^F-FMISO and Cu-ATSM had a good efficacy in tumor hypoxia imaging. ^18^F-FMISO was slowly accumulated in vivo and the image contrast of hypoxic areas was poor, while ^64^Cu-ATSM has several advantages, including a simple and rapid method for radiolabeling, a short time between injection and imaging, a better target to background ratio, an easy quantification method, and better image quality; despite a less favorable dosimetry, ^64^Cu-ATSM appears to be superior in terms of imaging performance [76].

## 7. Conclusions and Future Prospective

Angiogenesis is a complex biological process that plays a central role in progression of tumor growth and metastasis. There is great interest in agents against angiogenesis approved for therapeutic use in cancer patients, but it is very difficult to evaluate the treatment response of these anti-angiogenic drugs, because of their cytostatic, rather than cytotoxic, effect.

Molecular imaging may be helpful both for identification of malignant lesions, and for quantitative assessment of a specific target pathway involved in the angiogenic cascade. All the new radiotracers analyzed in the present review seem to have a potential role in diagnosis, staging, and follow up of cancer patients, however, further studies are needed.

These new tracers developed for tumor angiogenesis imaging can also have applications for other angiogenesis-related diseases, such as atherosclerosis, myocardial infarction, stroke, chronic inflammation, and many others.

## Figures and Tables

**Table 1:** Summary of radiopharmaceutical used for angiogenesis imaging.

Radiotracers	Imaging Mode	Biological Analog	Target Process	References
^18^F-FDG	PET	Glucose	Glucose metabolism, GLUT-1 expression	[16,17,18,19,20,21,22,23,24,25]
^123^I or ^125^I-VEGF165/121	SPECT	VEGF isoforms	VEGF pathway, bind to VEGFR	[26,27,28,29,30,31,32,33,34,35,36,37,38,39,40,41,42]
^99^mTc-VEGF121	SPECT	VEGF isoforms	VEGF pathway, bind to VEGFR-2	[42,43,44,45]
^111^In–VEGF165	SPECT	VEGF isoforms	VEGF pathway, bind to VEGFR	[46]
^64^Cu-VEGF121	PET	VEGF isoforms	VEGF pathway, bind to VEGFR-2	[47,48]
^111^In Bevacizumab	SPECT	VEGF	VEGF pathway; antibody against VEGF-A	[49]
^89^Zr Bevacizumab	PET	VEGF	VEGF pathway; antibody against VEGF-A	[49,50]
^125^I-RGD peptides	SPECT	Integrin αvβ3	Bind to RDG sequence of integrin	[51]
^18^F-Galacto RGD peptides	PET	Integrin αvβ3	Bind to RDG sequence of integrin	[51,52,53,54,55,56,57,58,59]
^68^Ga-NOTA-PRGD2	PET	Integrin αvβ3	Bind to RDG sequence of integrin	[55,59,60]
^64^Cu-DOTA RGDyK	PET	Integrin αvβ3	Bind to RDG sequence of integrin	[61,62]
^99^mTc-scFvL19	SPECT	Fibronectin	Fibronectin pathway, antibody against ED-B domain	[63,64]
^123^I-scFvL19	SPECT	Fibronectin	Fibronectin pathway, antibody against ED-B domain	[65]
^76^Br or ^124^I-L19 SIP	PET	Fibronectin	Fibronectin pathway, antibody against ED-B domain	[66,67]
^18^F-SAV 03M	PET	Matrix metalloproteinasis	Gelatinases pathway, inibithors of MMP-2	[68]
^68^Ga-NOTA-C6	PET	Matrix metalloproteinasis	Gelatinases pathway, inibithors of MMP-2 and 9	[69]
^18^F-FMISO	PET	Nitroimidazole	Hypoxia	[70,71,72,73,74]
^64^Cu-ATSM	PET		Hypoxia	[75,76]
